# Mechanics of reproductive differentiation in the land plants: a paradigm shift?

**DOI:** 10.3389/fpls.2024.1445582

**Published:** 2024-10-14

**Authors:** Philip M. Lintilhac

**Affiliations:** Department of Plant Biology, The University of Vermont, Burlington, VT, United States

**Keywords:** plant development, signaling pathways, stress-mechanics, reproductive differentiation, positional targeting, sporangial geometry, isotropic stress

## Abstract

This article addresses the physical mechanics of gametogenesis in vascular plants. The earliest events that initiate reproductive differentiation in the land plants are not well understood. How are the few cells that initiate reproductive differentiation specified and how is that information translated into action at the cellular level? In this article I propose a physical mechanism that resolves the problem of spatial targeting without invoking dependence on diffusible morphogens or other external signals. I suggest that the initiation of archesporial differentiation can instead be attributed to the confluence of organ geometry, surficial topography, and the physical mechanics of sporangial growth, resulting in the spontaneous emergence of an isotropic singularity that locates and precipitates archesporial differentiation. In discussing the logic of single-cell target selection and the limits of stochastic molecular signaling I propose that the sporangium would be better understood as a pressurized stress-mechanical lens that focuses turgor-generated growth forces on a central location, generating a physical singularity that locates and specifies the cell or cells that become the archesporium and initiates their transition from somatic proliferation to reproductive differentiation.

## Introduction

It is a surprising, and largely unappreciated fact that the key events leading to the differentiation of haploid gametes in the land plants are still not understood. Plant science has never been able to explain how select somatic cells at precise locations in the plant body can shift abruptly from vegetative proliferation to a developmental program committing them to meiosis, haploidization, and gametogenesis. Current thinking supposes that reproductive differentiation in the plant kingdom is, like many other aspects of biological development, prescribed by transcription-mediated molecular information networks and implemented through various molecular transport systems to specify a region of somatic cells as candidates for reproductive differentiation (Kelliher and Walbot, 2012a). But a fuller understanding of the architecture and mechanical continuity of plant tissues suggests that sporangial structure and geometry provide an ideal medium for deterministic, stress-mechanical signal propagation that can resolve and tag single-cells embedded in an otherwise undifferentiated multicellular mass.

## Background

In the animal kingdom reproductive differentiation occurs very early in life. Eggs and sperm, or their immediate precursors, differentiate in infancy. The resulting sexually competent cells are set aside as an independent, differentiated lineage that is maintained as the “reserved germ line” for the duration of reproductive life. The existence of a developmentally distinct germ line is familiar to us in that it makes it possible to harvest eggs and sperm for the modification and amplification of the natural breeding cycle, most notably for *in vitro* fertilization; but in plants there is no corresponding pre-determined lineage (Whipple, 2012; Russell and Jones, 2015; Nelms and Walbot, 2019; Twell, 2011). In plants, reproductive differentiation occurs late in the life cycle, unanticipated by any dedicated lineage that persists in the vegetative plant; because of this, gametes, or proto-gametes, cannot be harvested and used for plant breeding purposes as they can in the animal kingdom.

## Physical mechanics of plant growth

In the plant kingdom somatic cells are supported and constrained by an extensive scaffold of cell walls that provide structural support while preventing all cell movement and preserving nearest neighbor relationships. This continuous apoplastic scaffold provides a tissue-level mechanical coupling that makes physical signaling inevitable (Coen and Cosgrove, 2023). Mechanical forces propagate rapidly and accurately through turgid plant tissues, providing for the evolution of physical signaling that is instantaneous, spatially precise, and robust in the face of environmental perturbation. The observations that support this perspective on plant development are not new (Kwiatkowska and Nakielski, 2011). Physical interactions have been shown to play important roles in many aspects of plant morphogenesis (Echevin et al., 2019) and tissue patterning (Hamant and Louveaux, 2016; Nakayama et al., 2022; De Vos et al., 2012; Ali and Traas, 2016; Jiao et al., 2021). Physical information networks also overcome many of the inherent limitations of stochastic molecular information systems (Hemenway and Gehring, 2023; Kurusu et al., 2013; Lintilhac, 2022).

## Reproductive differentiation and sporangial structure

In the land plants reproductive differentiation and meiosis are restricted to specialized multicellular organs called sporangia which share many structural and geometrical features across all families of land plants (Goebel, 1905; Bower, 1935). Meiosis is initiated *de novo* by the abrupt differentiation of a single cell, or a small group of competent cells that are distinct from the somatic cells making up the rest of the vegetative plant body (Kelliher et al., 2014; Walbot and Egger, 2016).

**Figure 1:**
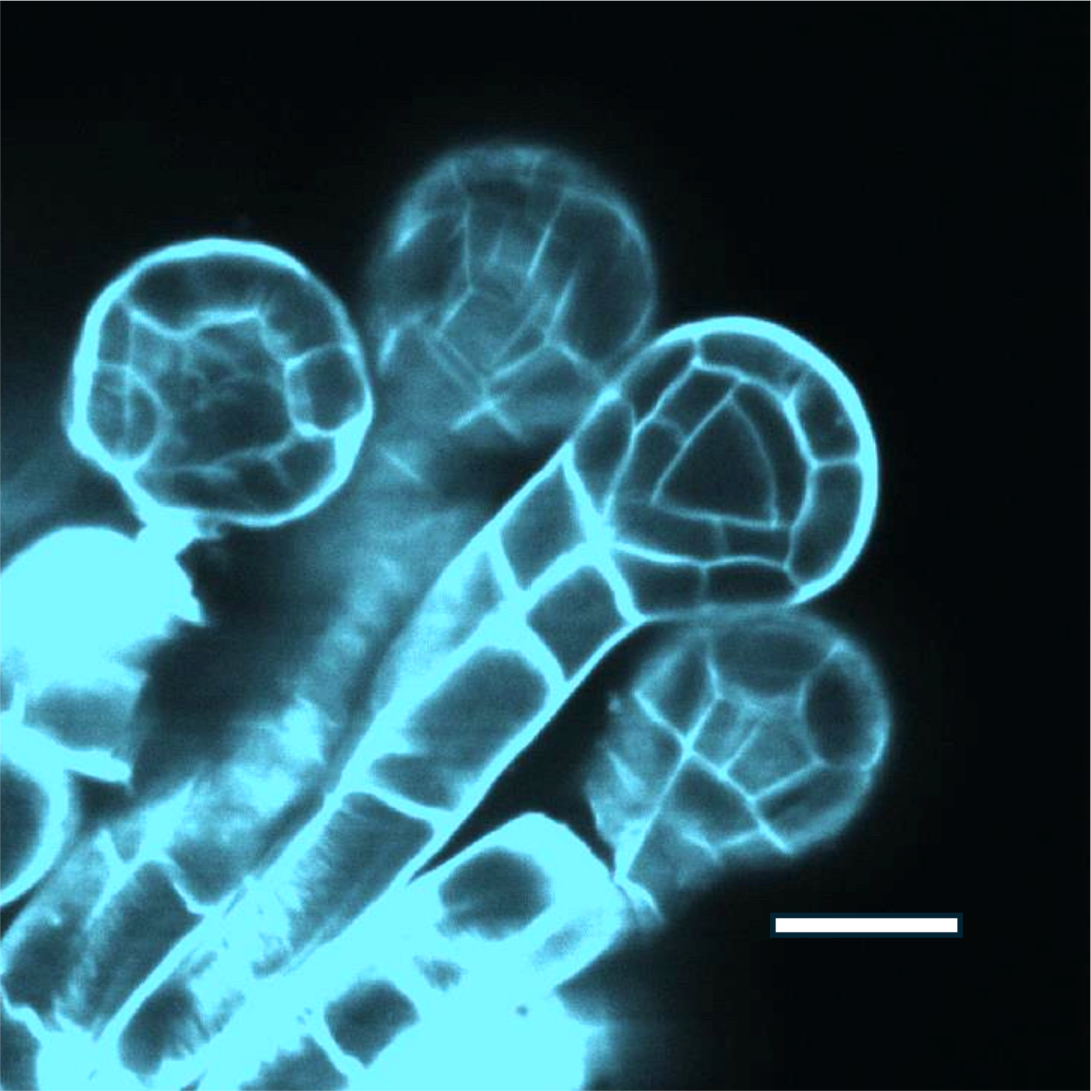
*Polypodium vulgare*, immature sporangia, cleared and stained with calcofluor white, showing the early tetrahedral archesporium. Andor Dragonfly confocal image. Scale bar 50u.

In the ferns early sporangial development consists of a sequence of stereotypical mitotic divisions and cell wall installations that build the form of the pre-meiotic sporangium, isolating a single centrally located archesporial cell that proceeds through meiosis and sporogenesis. (**Figure 1**).

In the flowering plants we find two sporangial morphologies, each dedicated to the initiation of either the male or the female germ lines. The most familiar of these is the anther sac, or microsporangium, within which an elongated axisymmetric archesporium differentiates, leading to meiosis and the production of sperm-producing pollen grains (microgametophytes). The female germ line originates in the nucellus, or megasporangium, which is formed in a parallel developmental sequence. Both the male and female developmental programs culminate at anthesis in the dispersal and transfer of pollen, and double-fertilization (Raghavan, 2003).

The first evidence of entry into the reproductive cycle is the differentiation of the archesporium, which consists of a single centrally located cell, or a small group of similarly competent cells, that are the first identifiable precursors to meiosis and the first evidence of a shift in developmental fate towards sexual reproduction.

The question is: What is the signal that initiates the selection and differentiation of the archesporium? What confluence of naturally occurring variables can identify and tag a single cell embedded in a mass of clonal neighbors at a precise moment in sporangial development without precipitating a similar developmental shift in any of its immediate neighbors?

## Positional effects

The salient attribute of this sequence of events is that the inducing stimulus must be highly location-specific, meaning that only the most centrally located cell (or cells) differentiates. Following induction, a cascade of developmental programs (Kelliher and Walbot, 2012b) is set in motion which channel the archesporial cells through the subsequent stages of reproductive differentiation and gametogenesis (Walbot and Egger, 2016).

In many of the ferns, as in the nucellus of the flowering plants, the archesporium consists of only a single cell, implying that the inducing signal must be able to identify a cell located at the geometric center of the sporangium. This means that the selective triggering of reproductive differentiation becomes a targeting problem. What kind of inducing stimulus can selectively specify a single, precisely located cell embedded in a cohort of many similarly constituted neighbors?

Positional effects in differentiating cellular systems have been explained in a variety of ways, perhaps most notably in the work of Lewis Wolpert, whose Positional Information Theory proposes that diffusible chemical species termed morphogens establish gradients across a field of cells, thereby discriminating one location from another by mapping local concentration signatures and enabling individual cells to follow location-specific protocols that determine their developmental fates (Wolpert, 1971). But there is limited direct evidence addressing the question of reproductive target selection in plants (Kelliher and Walbot, 2012a). The prevailing view shares many of the features of Wolpert’s Positional Information Theory, in that the selection process is presumed to be carried out by molecular and/or hormonal gradients.

But the intrinsically stochastic nature of molecular signaling systems is hard to reconcile with the observable specificity and robustness of archesporial specification in plants (Lintilhac, 2022). To put it simply, the immature sporangium at the time of archesporial specification is so small, and the number of likely cellular targets are so few, that an information system based on molecular population dynamics cannot provide the necessary spatial resolution. This suggests that there must be an alternate targeting protocol that does not depend upon molecular demographics or morphogen transport, but instead must depend upon deterministic signals that can reliably pinpoint a single cell at a precise location in a multicellular mass.

## The proposal

In the case of the growing premeiotic sporangium, physical and mechanical information systems have an advantage over morphogen-based systems because they are functionally deterministic and spatially precise, but also because they can encode the surface topography of the organ, reflecting the overall geometry of the structure while providing the resolution necessary to pinpoint, and tag, a single cell at a precise location without including any of its immediate neighbors. In more general terms, the governing variables must be able to establish a unique “singularity” that distinguishes one location from all others without regard to lineage.

According to this proposal the sporangium acts as a stress-mechanical focusing device that uses physical forces, generated by cell expansion growth and focused by organ geometry, to create a centrally located physical singularity that is robust and predictable, and does not depend upon the stochastic behavior of molecular populations. The growing sporangium provides the necessary physical shape and surface topography to focus stress fields on a precisely located central region. By using the strong mechanical coupling of neighboring plant cells, the force-generating capabilities of turgor-driven plant cell growth (Wolf, 2022; Hejnowicz, 2011), and by engaging the micro-architecture of the early sporangium, it is proposed that plants have evolved a robust physical/mechanical information system capable of reliably selecting individual cells for reproductive differentiation without relying on the stochastic behavior of molecular populations. Under this paradigm the signal that leads to the differentiation of the archesporium emerges spontaneously from the architecture and mechanics of the growing sporangium, independent of any other external inducing signal.

## Singularity and isotropy

**Figure 2:**
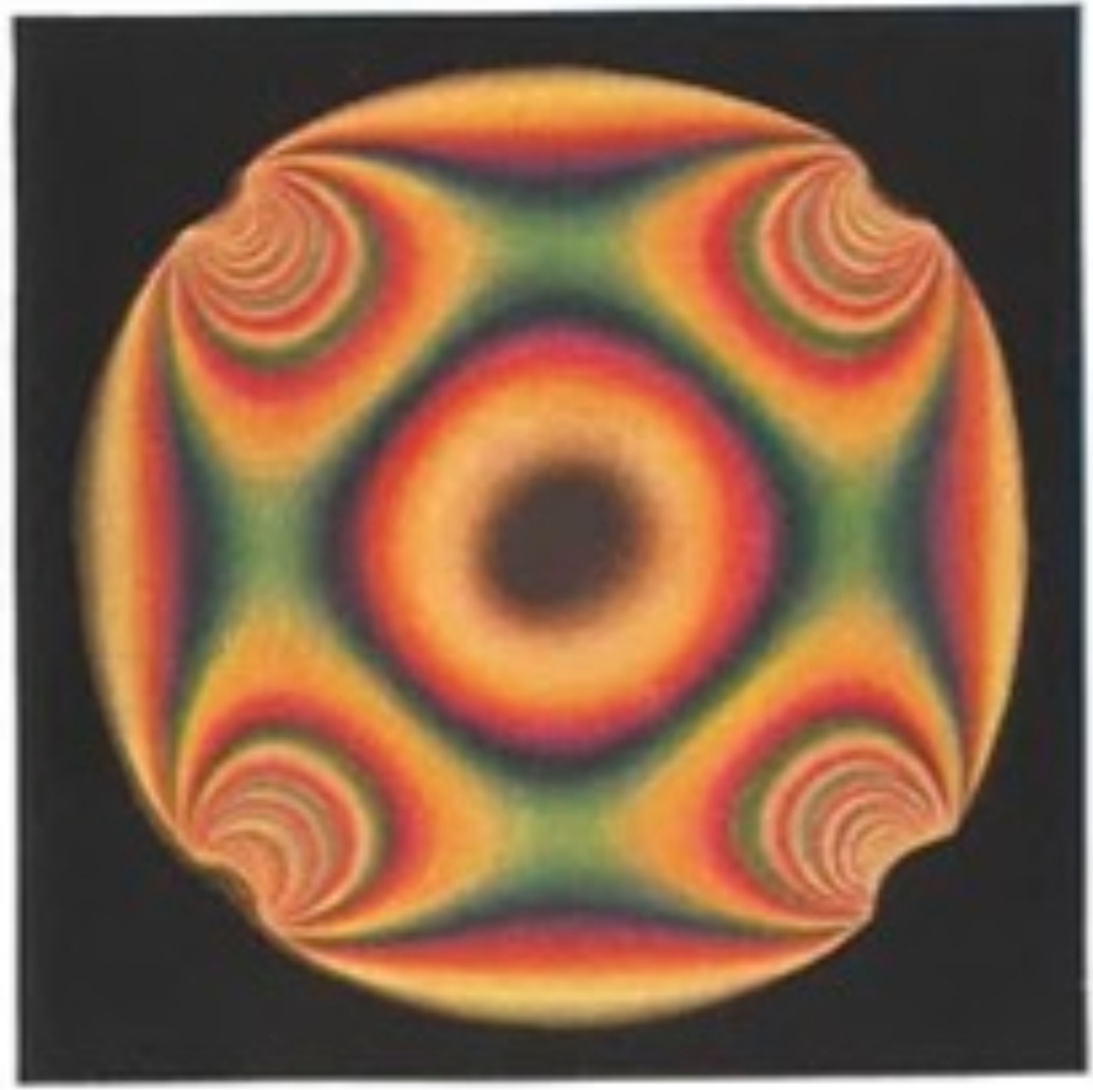
Photoelastic image of a polymer disk seen in circularly polarized light, subjected to 4 equal loads acting along mutually perpendicular diameters and producing an isotropic point at the center. After Frocht, 1941.

Physical singularities of this type are well documented in the historical engineering literature on stress analysis, where they are described as *isotropic points* (Frocht, 1941). Briefly, isotropy means that material properties are independent of direction. For instance, we can speak of material isotropy in polymers, where the structural orientation of the macromolecular constituents is the same in all directions, as in a fragment of cellulosic cell wall whose microfibrillar constituents are randomly oriented. We can also speak of optical isotropy, where optical retardation measured on any axis of polarization does not vary with the orientation of the polarizer. But we can also speak of stress-mechanical isotropy, where stress transmission at a given location in a solid shows no directionality. In fact, these different manifestations of physical isotropy are often linked, representing different views of the same phenomenon. In this sense isotropic points are distinguished from all other locations in a structure under load by their lack of any stress-mechanical directionality and the absence of all shear stress (Fung and Tong, 2001). In photoelastic materials they can be identified as localized regions of zero optical path difference and zero fringe order (**Figure 2**) (Frocht, 1941).

Isotropic points can also be diagrammed graphically using the method of *Mohr’s Circle of Stress* (Frocht, 1941). Mohr’s Circle has been described as a semi-graphical procedure (Muvdi and McNabb, 1980) for dissecting and reconstructing the stress-mechanical details of a structure under load. One outcome of the Mohr’s Circle procedure is that stress mechanical isotropy can be defined as any location where Mohr’s Circle collapses to a point of zero radius, indicating that all stress directionality is eliminated, and all shear stress vanishes.

## The controlling feedback

Mechanical stress (transmitted force) in living plant tissues and organs can arise either from externally applied loads like wind or gravity, or from internal sources such as cell enlargement and growth. In either case tensile and compressive stresses propagate through the surrounding tissue at the speed of sound. The resulting tensions and compressions can be represented as families of orthogonal principal stresses which follow straightforward rules as to their behavior at surfaces (Heywood, 1969). In actively dividing plant tissues and meristems this is often reflected in the geometric precision of cell wall installations in epidermal layers where stresses necessarily run parallel, and perpendicular to external surfaces.

During normal vegetative growth, and particularly in meristems, changes in meristem shape redirect and re-channel growth forces and result in a subsequent reconfiguration of tissue architecture. In other words, as meristem shape changes the physical cues orienting the next round of divisions are updated and the next iteration of morphogenetic form is locked in. This means that active meristems can to a certain extent be regarded as surface-generating automata that can be contrived to cycle through repeated iterations of surface shape change and subsurface tissue architecture.

The propensity for principal stress trajectories to be channeled by surface topography means that stress patterning can also be constrained by surface shape such that body stresses can in fact be configured to converge on a singular subsurface location, forming an isotropic point where all directionality cancels out, and which can be created or collapsed instantly by controlling the intensity of the transmitted forces at their source.

However, this brings us to a critical juncture in our understanding of cell behavior in growing plant structures, because if division orientations and cell expansion are tightly linked to stress directionality, then what does it mean when cells at the focus of an embedded isotropic singularity find themselves in an environment where there is a total lack of directional cues? Does this signal a fundamental change in cell behavior? How can cells maintain their normal mitotic polarity when the physical cues necessary to maintain that polarity cease to exist? Clearly, cells located at the focus of a stress-mechanical singularity cannot maintain the normal vegetative responsiveness to directional mechanical signals and may be forced to drop out of the normally polarized feedback cycle of vegetative proliferation. This localized breakdown in the normal cycle of oriented cell division and polarized growth explains the unique targeting potential of spherical and axisymmetric sporangial structures. The stress-mechanical singularity that emerges from this configuration is then available to serve as a unique inducing stimulus that can initiate a site-specific transition to reproductive differentiation. The downstream consequences of this structurally imposed shift in cell behavior have yet to be described, but the likelihood of it leading to a major divergence in developmental fate is undeniable.

In its simplest form then, the proposal put forward here suggests that in the evolution of the land plant sporangium, organ geometry and surface topography have combined with the force-generating abilities of turgid, growing, plant cells to locate and initiate the events that lead to sexual differentiation. By extension, it implies that the differentiation of all spores and gametes in the land plants depend upon the generation of localized regions of stress-mechanical isotropy that provide positionally specific physical cues, initiating a sequence of events that lead to reproductive differentiation at precise locations in growing sporangial structures.

The targeting ability of the sporangium can then be understood as an emergent property which arises spontaneously out of sporangial geometry and the inherent characteristics of plant growth, and which does not depend on stochastic molecular processes or other external signals to provide the positional cues that initiate pre-meiotic differentiation.

## Experimental approaches

Students of plant development are becoming increasingly aware of the influence of physical/mechanical stress on the behavior of growing plant tissues (Nakayama et al., 2022; Jackson et al., 2019), but one impediment to the experimental analysis of force transmission in any solid, cellular or otherwise, is that while internal body stresses can be represented rigorously in mathematical terms, they are in fact invisible to the naked eye. Real time stress directionalities and intensities can be revealed in many materials by using polarized light to follow stress-induced birefringence, (Heywood, 1969; Frocht, 1941), but the pronounced intrinsic birefringence of the plant cell wall makes polarized light methods difficult to implement in living plant tissues. The issue of visualizing stress and shear in living plant tissues is further complicated by the very small scale of the structures at the time of early reproductive differentiation. These observational issues make it difficult to verify the assumptions and predictions of modeling protocols such as Finite Element Modeling (FEM).

Direct measurement of stress directionality and intensity in tiny structures such as the immature sporangium, is currently beyond our ability. However, it may be possible to reproduce the physical configuration of the living sporangium in engineered structures capable of re-creating the stress-mechanical environment of the native sporangium, showing that manipulation of the physical environment can initiate reproductive differentiation, and opening a path to the experimental manipulation of germ line tissues *in vitro*. In an initial step toward this goal, we have shown that it is possible to use microfluidic droplet systems to isolate and capture single, living plant cells in hydrogel microspheres, potentially providing away to engineer synthetic isotropic stress-mechanical environments at the scale of the pre-meiotic sporangium (Grasso and Lintilhac, 2016).

The ultimate goal of such an effort would be to re-create the biophysical and biomechanical environment of the growing sporangium in a biomimetic structure that could be manipulated to control the flow of internal body stresses in a sporangium-like structure in order to initiate reproductive differentiation *in vitro*.

## Discussion

In the plant kingdom developmental ontogeny has evolved within the constraints of apoplastic continuity, immotile cells, and turgor-driven growth, and while the role of cell and tissue mechanics is correctly seen against a background of molecular control systems operating at the intracellular level, there are limits to the ability of molecular signals to coordinate precise structural decisions at the transcellular level. Information flows based on changes in molecular populations are necessarily stochastic, whereas physical signals can be configured to carry out actions at trans-cellular distances instantly, without relying on molecular transport at all. It appears that evolution has found ways to use physical information networks to manage key decisions in ways that are spatially precise, instantaneous, and robust, and can operate in environments where molecular systems cannot.

When one considers that physical force is classically deterministic in that it can produce action at a distance, instantly, and with a high degree of spatial accuracy with respect to the overall geometry of the sporangium itself, the advantages of targeting systems based on physical force transmission become clear. Mechanical information is rigorously constrained by external surfaces, taking on their attributes in very specific ways (Heywood, 1969). Furthermore, the flows of mechanical stress within a solid such as a cellular tissue can be switched on and off instantly by controlling the osmotic driving forces at their source, and while information networks based upon the localized distribution of molecular populations may be rich in terms of stereochemical information and selective binding affinities, they only weakly reflect the geometry and surface topography of the sporangium as a whole.

While the proposal outlined here is only preliminary, a new paradigm begins to emerge wherein the details of the stress-mechanical environment, tissue architecture, and surficial geometry of the growing organ, provide a continuously evolving landscape of structure-specific information which is seamlessly updated with changing shape, and which will not degrade with time as long as the driving forces persist (Khadka, 2020).

## Conclusion

Lastly, it is worth noting that our most important cereal crops and animal feeds are all propagated from seed, and every viable seed contains an embryo that can be traced back to the brief, singular, events that initiate the male and female germ lines. We need to understand this sequence of events in detail.

Unscripted physical networks appear to have evolved in parallel to the transcription-directed networks that are dominant at the intracellular level. Just as genetic scripting of the developmental process has evolved through the incremental accumulation of individual mutationally driven changes, and subjected to the test of survival, so too, the physical interactions that underlie tissue patterning and organ shape also reflect the incremental incorporation of diverse material behaviors that are assembled and concatenated by survival.

This article presents a new way of thinking about spatial signaling in plants; and while the hypothesis put forward here may be a step toward understanding how physical signals can resolve the location of positional targets the size of a single cell, it does not resolve the question of how an individual cell, or small groups of cells, can interpret these stress-mechanical singularities and translate them into terms that can be recognized and acted upon by cellular and sub-cellular processes. Clearly, the most likely candidate for this task would be the cytoskeleton, which is well equipped to measure dynamic changes in stress, strain, and shear at the cytoplasmic level (Louveaux and Hamant, 2013). Familiar cytoskeletal processes, perhaps exemplified by the pre-prophase band mechanism, would seem to be ideally suited to interpreting the stress-mechanical status of individual cells in a way that can be acted upon at the cytoplasmic level.

## Data Availability

The original contributions presented in the study are included in the article/supplementary material. Further inquiries can be directed to the corresponding author.
